# Sex-based differences in the anatomic distribution of cutaneous squamous cell carcinoma

**DOI:** 10.1016/j.ijwd.2020.05.008

**Published:** 2020-05-27

**Authors:** Yuhree Kim, Jessica Feng, Katherine A. Su, Maryam M. Asgari

**Affiliations:** a Department of Dermatology, Massachusetts General Hospital, Boston, MA, United States; b Department of Population Medicine, Harvard Pilgrim Health Care Institute, Boston, MA, United States

**Keywords:** Squamous cell carcinoma, Skin cancer, Gender disparity, Anatomic distribution, Epidemiology

## Abstract

**Background:**

Cutaneous squamous cell carcinoma (cSCC) is the second most common malignancy in the United States, and its incidence is increasing. Ultraviolet radiation is the main environmental risk factor for cSCCs; thus, they tend to arise on sun-exposed skin. Most publications cite the head and neck as the predominant location for cSCCs, but these papers do not account for the differential anatomic predication of cSCCs by sex. No prior studies have examined the differential distribution of cSCCs by sex, particularly invasive cSCCs that have the potential for recurrence and metastasis.

**Objective:**

We examined the association between cSCC tumor features, including anatomic site and invasiveness, by key patient features, including age and sex.

**Methods:**

Using an institutional cSCC registry, we identified 618 non-Hispanic white patients diagnosed with 2,111 histologically-confirmed cSCCs between 2000–2016.

**Results:**

We found differential anatomic distributions of cSCC by patient sex. Men were more likely to have cSCCs arise on the head and neck (51.7%), whereas women were more likely to have cSCCs develop on the lower extremity (41.2%). Stratification by dichotomized age (younger [<65 years] vs. older [≥65 years]) revealed that nearly half of invasive cSCCs (47.7%) among older women arose on the lower extremities, whereas approximately half of the invasive cSCCs (52.4%) arose on the head and neck among older men.

**Conclusion:**

Lower extremities can be easily overlooked, particularly when practitioners perform waist-up-only skin examinations in time-limited settings. Understanding the anatomic predilection for invasive cSCCs by patient characteristics, including our findings, which suggest that the lower extremities are an important anatomic site for invasive cSCCs among women, can help further inform skin cancer screening and prevention efforts.

## Introduction

Cutaneous squamous cell carcinoma (cSCC) is the second most common malignancy in the United States, and its incidence is increasing (Que et al., 2018). The majority of cSCCs are curable, particularly when treated at an early stage. However, invasive cSCCs with clinically aggressive features can reoccur and metastasize (Fox et al., 2019). In certain geographic regions of the United States, the number of deaths from invasive cSCC rivals that of melanoma (Karia et al., 2013), highlighting the need to focus screening and prevention efforts on identifying invasive cSCC. cSCCs tend to arise on sun-exposed skin. The most common anatomic distribution of cSCCs is often cited as the head and neck (English et al., 1998, Lim and Asgari, 2019), and the difference in anatomic distribution of the tumor by sex is often not highlighted. Delayed recognition of cSCC may lead to more advanced disease.

Although a full-body skin examination is the gold standard for skin cancer detection, many providers perform a limited skin examination (i.e., waist-up exams) only and may not examine lower extremities, mostly due to time constraints (Oliveria et al., 2011). Although differential anatomic distribution of cSCCs by sex has been reported (Kossard and Rosen, 1992, Subramaniam et al., 2017, Youl et al., 2011), to our knowledge, previous studies have not examined the anatomic predilection of invasive cSCCs by sex. Identification of anatomic sites at high risk for invasive cSCCs may inform screening and prevention efforts. The aim of our study was to assess the anatomic distribution of invasive cSCC by age and sex using an institutional cSCC registry to inform skin examinations that may lead to earlier detection of invasive cSCC and improve skin cancer outcomes.

## Methods

Using an established Biobank-based cSCC registry at Partners HealthCare (Kim et al., 2020), a large, U.S.-based medical center, we identified 618 non-Hispanic white patients with 2166 pathology-confirmed cSCCs between 2000 and 2016. The cSCC registry consists of individuals who received care at Partners HealthCare, a Boston-based non-profit hospital network including Massachusetts General Hospital, Brigham and Women’s Hospital, Faulkner Hospital, Newton-Wellesley Hospital, North Shore Medical Center, McLean Hospital, and Spaulding Rehabilitation Hospital.

We identified individuals who had a clinician-rendered International Classification of Disease (ICD) diagnosis code for cSCC (ICD 9th Revision, Clinical Modification codes 173.0–173.9, 186.7, 198.2, and Z85.828, or ICD 10th Revision, Clinical Modification codes C44.0-C44.9 and V1083) and who had an electronic pathology report that contained a cutaneous or skin-related text-string (skin or squamous or kerato) in the Partners Research Patient Data Registry (n = 5031). We excluded pathology reports from the review if they contained a noncutaneous text-string (prostate, esophagus, gastric, stomach, colon, marrow, urothelial, cervical, urine, bone, or cerebrospinal). Two trained abstractors (JF and KS) validated the cSCC diagnosis by reviewing electronic pathology reports and abstracting tumor features, including anatomic site (categorized as head and neck, upper extremity, lower extremity, trunk, and buttocks) and invasiveness (in situ vs. invasive). We excluded cSCCs arising on the lip (n = 35) and genitals (n = 16), as well as those with unknown anatomic sites (n = 4), leaving 2111 cSCCs for analysis. Age was defined at incident cSCC diagnosis date. A χ^2^ test was used to compare categorical variables. All analyses were performed with STATA, version 15.0 (StataCorp, College Station, TX). This study was approved by the Partners Healthcare Institutional Review Board.

## Results

Of the 618 patients, 366 were male (59.2%), consistent with previously reported male sex predilection. The mean follow-up of the study cohort was 5.1 years. Head and neck was the most common anatomic site for overall tumors (n = 849; 40.2%), followed by upper extremity (n = 537; 25.4%) and lower extremity (n = 451; 21.4%; Table 1). Anatomic distribution differed by sex with head and neck as the most common anatomic site among men (n = 685; 51.7%) and lower extremity as the most common site among women (n = 324; 41.2%). More cSCCs arose on the buttocks among women compared with men (2.6% vs. 0.1%; *p* < .001). When stratified by tumor invasiveness, nearly half of invasive cSCCs arose on the lower extremities among women (n = 210; 44.0%), and a smaller fraction (n = 73; 15.3%) arose on the head and neck (Table 1). In contrast, the majority of invasive cSCCs arose on the head and neck among men (n = 439; 53.8%), and a smaller fraction (n = 91; 11.2%) arose on the lower extremities. Of note, 14 invasive cSCCs (2.9%) arose on the buttocks among women. For all other sites, there was no statistically significant difference between women and men.

**Table 1:** Variations in anatomic distribution of cutaneous squamous cell carcinoma by patient age and sex and tumor invasiveness.

	Overall								Invasiveness							
									In situ				Invasive			
Location	Total		Female		Male		*p*-value		Total	Female	Male	*p*-value	Total	Female	Male	*p*-value
	n (%)		n (%)		n (%)				n (%)	n (%)	n (%)		n (%)	n (%)	n (%)	
Head and neck^a^	849 (40.2)		164 (20.9)		685 (51.7)		<0.001		337 (41.2)	91 (29.5)	246 (48.3)	<0.001	512 (39.6)	73 (15.3)	439 (53.8)	<0.001
Upper extremities^b^	537 (25.4)		192 (24.4)		345 (26.0)		0.412		210 (25.7)	70 (22.6)	140 (27.5)	0.124	327 (25.3)	122 (25.6)	205 (25.1)	0.856
Trunk^c^	253 (12.0)		86 (10.9)		167 (12.6)		0.256		115 (14.1)	28 (9.1)	87 (17.1)	0.001	138 (10.7)	58 (12.2)	80 (9.8)	0.186
Buttocks	21 (1.0)		20 (2.6)		1 (0.1)		<0.001		6 (0.7)	6 (1.9)	0 (0)	0.002	15 (1.2)	14 (2.9)	1 (0.1)	<0.001
Lower extremities^d^	451 (21.4)		324 (41.2)		127 (9.6)		<0.001		150 (18.3)	114 (36.9)	36 (7.1)	<0.001	301 (23.3)	210 (44.0)	91 (11.2)	<0.001
	Age < 65 years								Age ≥ 65 years							



	In situ				Invasive				In situ				Invasive			

Location	Total	Female	Male	*p*-value	Total	Female	Male	*p*-value	Total	Female	Male	*p*-value	Total	Female	Male	*p*-value
	n (%)	n (%)	n (%)		n (%)	n (%)	n (%)		n (%)	n (%)	n (%)		n (%)	n (%)	n (%)	

Head and neck^a^	105 (41.5)	23 (27.1)	82 (48.8)	0.001	147 (39.1)	22 (14.1)	125 (56.8)	<0.001	232 (41.1)	68 (30.4)	164 (48.1)	<0.001	365 (39.8)	51 (15.9)	314 (52.7)	<0.001
Upper extremities^b^	70 (27.7)	18 (21.2)	52 (30.9)	0.101	93 (24.7)	41 (26.3)	52 (23.7)	0.558	140 (24.8)	52 (23.2)	88 (25.8)	0.485	234 (25.5)	81 (25.2)	153 (25.7)	0.885
Trunk^c^	34 (13.4)	7 (8.2)	27 (16.1)	0.084	52 (13.8)	24 (15.4)	28 (12.7)	0.462	81 (14.3)	7 (8.2)	60 (17.6)	0.006	86 (9.4)	34 (10.6)	52 (8.7)	0.355
Buttocks	6 (2.4)	6 (7.0)	0 (0)	<0.001	12 (3.2)	12 (7.7)	0 (0)	<0.001	0 (0)	0 (0)	0 (0)	–	3 (0.3)	2 (0.6)	1 (0.2)	0.249
Lower extremities^d^	38 (15.0)	31 (36.5)	7 (4.2)	<0.001	72 (19.2)	57 (36.5)	15 (6.8)	<0.001	112 (19.8)	83 (37.0)	29 (8.5)	<0.001	229 (25.0)	153 (47.7)	76 (12.7)	<0.001

a Head and neck includes the face, eyelids, scalp, ears, and neck.

b Upper extremities includes the arms, hands, and shoulders.

c Trunk includes the back, chest, and abdomen.

d Lower extremities includes the thighs, legs, and feet.

We found similar results when we further stratified our results by dichotomized age categories (younger [<65 years] vs. older [≥65 years]; Table 2). More than one-third of invasive cSCCs (n = 57; 36.5%) among younger women and nearly half of invasive cSCCs (n = 153, 47.7%) among older women arose on the lower extremities, and a smaller fraction arose on the head and neck (n = 22; 14.1% among younger women; n = 51; 15.9% among older women). In contrast, the majority of invasive cSCCs in younger (n = 125; 56.8%) and older men (n = 314; 52.4%) arose on the head and neck, and a much smaller fraction (n = 15; 6.8% among younger men; n = 76; 12.6% among older men) arose on the lower extremities. cSCCs on the lower extremities were more likely to be invasive in women (44.0% invasive vs. 36.9% in situ; *p* = .047) and men (11.2% invasive vs. 7.1 in situ%; *p* = .014). The risk of invasiveness became more prominent with older age (47.7% invasive vs. 37.0% in situ among older women and 12.7% invasive vs. 8.5% in situ among older men).

**Table 2:** Variations in anatomic distribution of cutaneous squamous cell carcinoma by patient sex, age, and tumor invasiveness.

	Female								Male							
Location	Total		In-situ		Invasive		*p-value*		Total		In-situ		Invasive		*p-value*	
	n (%)		n (%)		n (%)				n (%)		n (%)		n (%)			
Head and neck^a^	164 (20.9)		91 (29.5)		73 (15.3)		<0.001		685 (51.7)		246 (48.3)		439 (53.8)		0.053	
Upper extremities^b^	192 (24.4)		70 (22.6)		122 (25.6)		0.352		345 (26.0)		140 (27.5)		205 (25.1)		0.336	
Trunk^c^	86 (10.9)		28 (9.1)		58 (12.2)		0.174		167 (12.6)		87 (17.1)		80 (9.8)		<0.001	
Buttocks	20 (2.6)		6 (1.9)		14 (2.9)		0.388		1 (0.1)		0 (0)		1 (0.1)		0.429	
Lower extremities^d^	324 (41.2)		114 (36.9)		210 (44.0)		0.047		127 (9.6)		36 (7.1)		91 (11.2)		0.014	
	Age < 65 years								Age ≥ 65 years							
	Female				Male				Female				Male			

Location	Total	In situ	Invasive	*p*-value	Total	In situ	Invasive	*p*-value	Total	In situ	Invasive	*p*-value	Total	In situ	Invasive	*p*-value

n (%)	n (%)	n (%)	n (%)	n (%)	n (%)	n (%)	n (%)	n (%)	n (%)	n (%)	n (%)

Head and neck^a^	45 (18.7)	23 (27.1)	22 (14.1)	0.014	207 (53.3)	82 (48.8)	125 (56.8)	0.117	119 (21.8)	68 (30.4)	51 (15.9)	<0.001	478 (51.0)	164 (48.1)	314 (52.7)	0.176
Upper extremity^b^	59 (24.5)	18 (21.2)	41 (26.3)	0.378	104 (26.8)	52 (30.9)	52 (23.7)	0.107	133 (24.4)	52 (23.2)	81 (25.2)	0.589	241 (25.7)	88 (25.8)	153 (25.7)	0.964
Trunk^c^	31 (12.8)	7 (8.2)	24 (15.4)	0.113	55 (14.2)	27 (16.1)	28 (12.7)	0.349	55 (10.1)	21 (9.4)	34 (10.6)	0.643	112 (12.0)	60 (17.6)	52 (8.7)	<0.001
Buttocks	18 (7.5)	6 (7.0)	12 (7.7)	0.858	0 (0)	0 (0)	0 (0)	–	2 (0.4)	0 (0)	2 (0.6)	0.237	1 (0.1)	0 (0)	1 (0.2)	0.449
Lower extremity^d^	88 (36.5)	31 (36.5)	57 (36.5)	0.992	22 (5.7)	7 (4.2)	15 (6.8)	0.263	236 (43.3)	83 (37.0)	153 (47.7)	0.014	105 (11.2)	29 (8.5)	76 (12.7)	0.047

a Head and neck includes the face, eyelid, scalp, ears, and neck.

b Upper extremities includes the arms, hands, and shoulders.

c Trunk includes the back, chest, and abdomen.

d Lower extremities includes the thighs, legs, and feet.

## Discussion

This retrospective cohort study found that head and neck was the most common anatomic site of cSCC among men, and the lower extremity was the most common site of cSCC among women. When stratified by invasiveness, the majority of invasive cSCCs among men arose on the head and neck, whereas approximately half of invasive cSCCs among women arose on the lower extremity. When stratified by both age and sex, cSCCs on the lower extremities were more likely to be invasive than in situ among older women and older men. To the best of our knowledge, this is the first study to stratify cSCC anatomic distribution by tumor invasiveness, an important prognostic factor that has been overlooked in prior studies of cSCC anatomic distribution, along with age and sex.

Highly cited clinical reference manuals, such as UpToDate, cite the head and neck as the most common anatomic site for cSCCs (Lim and Asgari, 2019). However, these findings are based on the literature, which does not take into account differential anatomic predication by sex. Of note, previous studies reported that cSCCs on the lower extremities occurred more commonly in women (Kim et al., 2014), and a recent study reported that the proportion of cSCCs on the extremities increased in women over the past several decades (Muzic et al., 2017). Our findings confirm and expand on the results of previous studies of the anatomic distribution of cSCCs.

Potential mechanisms for sex-specific patterns of the risk of cSCC development have not yet been studied extensively. Patterns of sex-specific lifestyle or behavior may explain the high incidence of cSCCs on the lower extremities in women, including clothing, tanning, and moisturizer use that may increase the skin’s exposure and sensitivity to ultraviolet radiation. Also, given that cSCCs have been associated with chronic inflammation, depilation of the lower extremities among women, including frequent shaving, chemical depilatories, waxing, or threading, may lead to chronic inflammation that could increase cSCC risk. In addition, differential exposure to sex-related hormones (e.g., estrogen) may affect the sex-specific development of cSCCs. Previous studies have reported that keratinocytes are estrogen-responsive cells with high estradiol-binding affinity, and exogenous estrogen use from oral contraceptives has been associated with increased cSCC risk (Asgari et al., 2010, Verdier-Sevrain et al., 2004).

Knowledge of cSCC anatomic distribution is particularly critical for invasive tumors, which can cause local tissue damage, recur, and potentially metastasize. The lower extremities are often categorized as an intermittently sun-exposed site (GTEx Consortium, 2015) and may not be a focal point for skin cancer screening, especially because the presence of clothing, such as pants, makes quick visual assessments difficult. Performing full-body skin examinations to detect cSCC represents a significant workload for health care providers, mostly due to time constraints, competing morbidities, and patient embarrassment (Oliveria et al., 2011). A previous study reported that about half of family practitioners or internists and approximately 20% of dermatologists do not routinely perform full-body skin examinations (Oliveria et al., 2011). Other studies reported that women were more likely than men to report feeling embarrassed by a full-body skin examination and therefore may be less likely to have full-body skin examinations performed, especially by sex nonconcordant providers (Federman et al., 2006, Houston et al., 2016, Rodriguez et al., 2007). Our findings highlight the need to closely examine the lower extremities of women and the head and neck of men, given that these are the most common sites of invasive cSCC.

The strengths of this study include the use of a large, Biobank-based registry with pathology-confirmed diagnoses. Several limitations should be considered when interpreting our results, including surveillance bias and utilization of a single-institution cohort, potentially limiting generalizability to other patient populations. Lastly, we could not rule out anatomic misclassification (e.g., some tumors classified as buttocks on the pathology report may, in fact, be perianal tumors that could have met exclusion criteria). However, the number of such potentially misclassified tumors is small. It is unlikely that most tumors, which arose on the head and neck and lower extremities, were misclassified.

## Conclusion

Knowing the anatomic site of predilection for cSCCs, particularly invasive tumors, by patient factors, such as age and sex, may inform skin cancer examinations and improve invasive cSCC detection and skin cancer outcomes. Furthermore, understanding variations in the anatomic distribution of in situ and invasive cSCC may provide insight into differential etiologies and prompt further research into other risk factors for cSCC, particularly those arising on anatomic sites traditionally thought of as intermittently sun-exposed, such as the lower extremities.

## Conflict of Interest

None.

## Funding

NA.

## Study Approval

The author(s) confirm that any aspect of the work covered in this manuscript that has involved human patients has been conducted with the ethical approval of all relevant bodies.

## Financial Disclosures

Dr. Asgari receives grant funding from Pfizer, Inc. for research studies unrelated to the subject matter addressed in this manuscript.

## References

[b0005] Asgari M.M., Efird J.T., Warton E.M., Friedman G.D. (2010). Potential risk factors for cutaneous squamous cell carcinoma include oral contraceptives: Results of a nested case-control study. Int J Environ Res Public Health.

[b0010] English D.R., Armstrong B.K., Kricker A., Winter M.G., Heenan P.J., Randell P.L. (1998). Demographic characteristics, pigmentary and cutaneous risk factors for squamous cell carcinoma of the skin: a case-control study. Int J Cancer.

[b0015] Federman D.G., Kravetz J.D., Haskell S.G., Ma F., Rirsner R.S. (2006). Full-body skin examinations and the female veteran: prevalence and perspective. Arch Dermatol.

[b0020] Fox M., Brown M., Golda N., Goldberg D., Miller C., Pugliano-Mauro M. (2019). Nodal staging of high-risk cutaneous squamous cell carcinoma. J Am Acad Dermatol.

[b0025] GTEx Consortium (2015). Human genomics. The Genotype-Tissue Expression (GTEx) pilot analysis: multitissue gene regulation in humans. Science.

[b0030] Houston N.A., Secrest A.M., Harris R.J., Mori W.S., Eliason M.J., Phillips C.M. (2016). Patient preferences during skin cancer screening examination. JAMA Dermatol.

[b0035] Karia P.S., Han J., Schmults C.D. (2013). Cutaneous squamous cell carcinoma: estimated incidence of disease, nodal metastasis, and deaths from disease in the United States, 2012. J Am Acad Dermatol.

[b0040] Kim C., Ko C.J., Leffell D.J. (2014). Cutaneous squamous cell carcinomas of the lower extremity: a distinct subset of squamous cell carcinomas. J Am Acad Dermatol.

[b0045] Kim Y., Su K.A., Asgari M.M. (2020). Correlates of multiple cutaneous squamous cell carcinoma: a retrospective cohort study. J Am Acad Dermatol.

[b0050] Kossard S., Rosen R. (1992). Cutaneous Bowen's disease. An analysis of 1001 cases according to age, sex, and site. J Am Acad Dermatol.

[b0055] Lim JL, Asgari MM. Clinical features and diagnosis of cutaneous squamous cell carcinoma (SCC). UpToDate: Waltham, MA. 2019.

[b0060] Muzic J.G., Schmitt A.R., Wright A.C., Alniemi D.T., Zubair A.S., Olazagasti Lourido J.M. (2017). Incidence and trends of basal cell carcinoma and cutaneous squamous cell carcinoma: a population-based study in Olmsted County, Minnesota, 2000 to 2010. Mayo Clin Proc.

[b0065] Oliveria S.A., Heneghan M.K., Cushman L.F., Ughetta E.A., Halpern A.C. (2011). Skin cancer screening by dermatologists, family practitioners, and internists: Barriers and facilitating factors. Arch Dermatol.

[b0070] Que S.K.T., Zwald F.O., Schmults C.D. (2018). Cutaneous squamous cell carcinoma: Incidence, risk factors, diagnosis, and staging. J Am Acad Dermatol.

[b0075] Rodriguez G.L., Ma F., Federman D.G., Rouhani P., Chimento S., Multach M. (2007). Predictors of skin cancer screening practice and attitudes in primary care. J Am Acad Dermatol.

[b0080] Subramaniam P., Olsen C.M., Thompson B.S., Whiteman D.C., Neale R.E., Qskin Sun and Health Study Investigators (2017). Anatomical distributions of basal cell carcinoma and squamous cell carcinoma in a population-based study in Queensland, Australia. JAMA Dermatol.

[b0085] Verdier-Sevrain S., Yaar M., Cantatore J., Traish A., Gilchrest B.A. (2004). Estradiol induces proliferation of keratinocytes via a receptor mediated mechanism. FASEB J.

[b0090] Youl P.H., Janda M., Aitken J.F., Del Mar C.B., Whiteman D.C., Baade P.D. (2011). Body-site distribution of skin cancer, pre-malignant and common benign pigmented lesions excised in general practice. Br J Dermatol.

